# Comparison of different tube-of-response (TOR) models for resolution recovery in PET image reconstruction for the Philips Ingenuity TF PET/MR

**DOI:** 10.1186/2197-7364-2-S1-A41

**Published:** 2015-05-18

**Authors:** Alexandr Lougovski, Frank Hofheinz, Jorg Van Den Hoff

**Affiliations:** Helmholtz-Center Dresden-Rossendorf, Institute of Radiopharmaceutical Cancer Research, PET Center, Dresden, Germany

Recently, we have proposed a method for on-the-fly system matrix computation where the tube-of-response (TOR) is approximated as a cylinder with constant density (TORCD) and the cubic voxels are replaced by spheres. We could show that with this model the PET image quality can be notably improved compared to the vendor provided image reconstruction of our Philips Ingenuity-TF PET/MR. In this work we address the question whether image quality can be further improved by using a variable density TOR (TOR-VD). The radial variability of TOR-VD was modelled by a Kaiser-Bessel function. Free parameters of this density model were used to optimize image properties regarding resolution, noise, and Gibbs artifacts. Additional, a TOR-VD model accounting for position dependent effects along the TOR caused by the finite solid angles of the detectors is under investigation. Phantom measurement were performed with a Philips Ingenuity-TF PET/MR scanner. Listmode data were reconstructed using TOR-CD and TORVD, respectively on two different grids with cubic voxel size of 2 mm and 4 mm. Image quality was assessed with resolution-noise curves and investigation of the radial position dependence of the spatial resolution. For 2 mm voxels, TOR-VD consistently yields a slight improvement of the investigated image quality measures compared to TOR-CD. For 4 mm voxels both models lead essentially to the same results. These findings can be understood as a consequence of the relative size of voxel and TOR. For typical whole body studies (4 mm voxel size) a variable TOR does not improve image quality beyond what is achievable with a constant density TOR. For smaller voxel size the image quality can indeed be somewhat improved with a variable TOR but at the expense of drastically increased computation time.

